# Depressive symptoms and risk of liver-related mortality in individuals with hepatitis B virus infection: a cohort study

**DOI:** 10.1038/s41598-020-77886-2

**Published:** 2020-11-30

**Authors:** In Young Cho, Yoosoo Chang, Eunju Sung, Won Sohn, Jae-Heon Kang, Hocheol Shin, Seungho Ryu

**Affiliations:** 1 Department of Family Medicine, Kangbuk Samsung Hospital, Sungkyunkwan University School of Medicine, 29 Saemunan-ro, Jongno-gu, Seoul, 03181 South Korea; 2 Center for Cohort Studies, Total Healthcare Center, Kangbuk Samsung Hospital, Sungkyunkwan University School of Medicine, Seoul, South Korea; 3 Department of Occupational and Environmental Medicine, Kangbuk Samsung Hospital, Sungkyunkwan University School of Medicine, Samsung Main Building B2, 250 Taepyung-ro 2ga, Jung-gu, Seoul, 04514 South Korea; 4 Department of Clinical Research Design and Evaluation, SAIHST, Sungkyunkwan University, Seoul, South Korea; 5 Division of Gastroenterology, Department of Internal Medicine, Kangbuk Samsung Hospital, Sungkyunkwan University School of Medicine, Seoul, South Korea

**Keywords:** Psychology, Gastroenterology

## Abstract

The impact of depression on the risk of liver-related mortality in individuals with hepatitis B virus (HBV) infection remains unclear. We examined the association between depression, HBV infection, and liver-related mortality. A total of 342,998 Korean adults who underwent health examinations were followed for up to 7.8 years. Depressive symptoms were defined as a Center for Epidemiologic Studies-Depression score ≥ 16. Cox proportional hazard models were used to estimate adjusted hazard ratios (aHRs) and 95% confidence intervals (CIs). During 1,836,508 person-years of follow-up, 74 liver-related deaths and 54 liver cancer deaths were identified (liver-related mortality rate of 4.0 per 10^5^ person-years and liver cancer mortality rate of 2.9 per 10^5^ person-years). Subjects with depressive symptoms had an increased risk of liver-related mortality with a corresponding multivariable aHR of 2.00 (95% CI 1.10–3.63) compared to those without depressive symptoms. This association was more evident in HBsAg-positive participants with a corresponding multivariable aHR of 4.22 (95% CI 1.81–9.88) than HBsAg-negative participants (P for interaction by HBsAg positivity = 0.036). A similar pattern was observed in relation to liver cancer mortality. In this large cohort, depressive symptoms were associated with an increased risk of liver-related mortality, with a stronger association in HBsAg-positive individuals.

## Introduction

Depression is an important public health concern due to its high lifetime prevalence, and is recognized as a leading cause of disability^1,2^. In addition to its disease burden, depression is also associated with increased overall mortality and disease-specific mortality among communities or patients with various illnesses^3,4^.

Liver disease accounts for about 2 million deaths per year globally, 1 million due to cirrhosis and 1 million due to viral hepatitis and hepatocellular carcinoma (HCC)^5^. Chronic hepatitis B virus (HBV) infection is also a global health problem that causes substantial liver-related morbidity and mortality^6,7^. While depression is frequently accompanied by other chronic diseases and can worsen their course^2^, individuals with HBV infection have been reported to have higher rates of depressive symptoms than healthy controls^8–10^. However, the impact of depression on liver-related outcomes, such as liver cancer and liver-related mortality, in individuals with HBV infection remains unknown.

Previous studies demonstrated a positive relationship between depression and liver-related mortality in specific populations such as liver transplant recipients and liver cancer patients^11,12^. In contrast, general population-based studies are scarce, and have reported conflicting findings^13,14^. Furthermore, no previous studies have examined the association between depression, liver cancer, and liver-related mortality in the general population according to the presence or absence of HBV infection.

Therefore, in the present study, we examined whether depressive symptoms are associated with liver-related mortality among hepatitis B surface antigen (HBsAg)-negative and HBsAg-positive individuals, while accounting for time-dependent measures of change in depressive symptoms and other covariates during follow-up.

## Results

At baseline, the mean (standard deviation) age of study participants was 39.6 (9.7) years, and 53.7% of participants were male (Table 1). A total of 10,834 (3.16%) participants were positive for HBsAg and 42,508 (12.4%) participants had depressive symptoms. Participants with depressive symptoms were more likely to be younger and female. When age and sex were adjusted for, depressive symptoms were positively associated with current smoking, alcohol intake, lower education level, obesity, diabetes, hypertension, total calorie intake, and slightly elevated levels of glucose, triglycerides, and homeostatic model assessment-insulin resistance (HOMA-IR). Depressive symptoms were also positively associated with medication for liver disease, fatty liver, and slightly higher levels of liver enzymes and Fibrosis-4 score (FIB-4). Participants with depressive symptoms at baseline were more likely to have a history of physician-diagnosed depression and current use of antidepressants.

**Table 1:** Estimated^†^ mean values (95% confidence intervals) and adjusted^†^ proportions (95% confidence intervals) of baseline characteristics of study participants according to CES-D score category.

Characteristics	CES-D score category		*p* value
	< 16	≥ 16	
Number	300,490	42,508	
Age (years)	39.7 (39.7–39.7)	38.8 (38.7–38.9)	< 0.001
Sex, male (%)	56.3 (56.1–56.5)	35.7 (35.3–36.2)	< 0.001
Body mass index (kg/m^2^)	23.3 (23.3–23.3)	23.4 (23.3–23.4)	< 0.001
Current smoker (%)	21.6 (21.4–21.7)	27.7 (27.3–28.2)	< 0.001
Alcohol intake (%)^‡^	23.1 (23.0–23.3)	29.8 (29.4–30.3)	< 0.001
Health-enhancing physically active (%)	16.4 (16.3–16.5)	15.9 (15.6–16.3)	0.021
High education level (%)^§^	80.5 (80.4–80.6)	72.3 (71.9–72.7)	< 0.001
Obesity (%)^¶^	28.0 (27.8–28.1)	29.0 (28.6–29.5)	< 0.001
Diabetes (%)	4.2 (4.2–4.3)	4.9 (4.7–5.1)	< 0.001
Hypertension (%)	11.7 (11.6–11.8)	12.7 (12.4–13.0)	< 0.001
History of CVD (%)	1.21 (1.17–1.25)	1.75 (1.62–1.88)	< 0.001
HBsAg positivity (%)	3.2 (3.1–3.2)	3.0 (2.8–3.2)	0.063
Family history of cancer (%)	26.8 (26.7–27.0)	27.1 (26.7–27.5)	0.251
Medication for liver disease (%)	1.4 (1.3–1.4)	1.9 (1.7–2.0)	< 0.001
Liver cirrhosis (%)	0.03 (0.03–0.048)	0.03 (0.01–0.05)	0.898
Fatty liver (%)	28.4 (28.2–28.5)	29.4 (29.0–29.8)	< 0.001
History of depression (%)	0.5 (0.5–0.5)	1.4 (1.3–1.5)	< 0.001
Use of an antidepressant (%)	0.2 (0.2–0.3)	1.6 (1.4–1.7)	< 0.001
Systolic blood pressure (mmHg)	109.3 (109.2–109.3)	109.0 (108.9–109.2)	0.001
Diastolic blood pressure (mmHg)	70.0 (70.0–70.1)	70.0 (69.9–70.1)	0.838
Glucose (mg/dL)	95.2 (95.2–95.3)	95.6 (95.5–95.8)	< 0.001
Total cholesterol (mg/dL)	193.7 (193.6–193.8)	193.7 (193.4–194.1)	0.971
LDL-C (mg/dL)	120.5 (120.4–120.6)	120.0 (119.7–120.3)	0.002
HDL-C (mg/dL)	58.9 (58.8–58.9)	58.9 (58.8–59.0)	0.865
Triglycerides (mg/dL)	110.4 (110.1–110.6)	112.9 (112.2–113.6)	< 0.001
Aspartate aminotransferase (U/L)	22.3 (22.3–22.4)	22.6 (22.5–22.8)	0.001
Aalanine aminotransferase (U/L)	23.6 (23.5–23.6)	24.0 (23.7–24.2)	0.055
Gamma-glutamyl transferase (U/L)	30.9 (30.7–31.0)	33.1 (32.7–33.4)	< 0.001
Fibrosis-4 score	0.820 (0.819–0.821)	0.822 (0.819–0.826)	< 0.001
HOMA-IR	1.47 (1.46–1.47)	1.52 (1.51–1.54)	< 0.001
Total calorie intake (kcal/day)^††^	1085 (1082–1089)	1125 (1117–1134)	< 0.001

*CVD* Cardiovascular disease, *HBsAg* hepatitis B surface antigen, *HDL-C* high-density lipoprotein-cholesterol, *HOMA-IR* homeostasis model assessment of insulin resistance, *LDL-C* low-density lipoprotein cholesterol, *CES-D* center for epidemiologic studies-depression.

^†^Adjusted for age and sex.

^‡^ ≥ 20 g of ethanol per day; ^§^ ≥ college graduate.

^¶^Body mass index ≥ 25 kg/m^2^.

^††^Among 237,504 participants with a plausible estimated energy intake level (within three standard deviations of the log-transformed mean energy intake).

During 1,836,508.5 person-years of follow-up, 74 liver-related deaths and 54 liver cancer deaths were identified (liver-related mortality rate of 4.0 per 10^5^ person-years and liver cancer mortality rate of 2.9 per 10^5^ person-years). The median duration of follow-up was 5.6 years (interquartile range, 3.9–6.8 years and maximum, 7.8 years). Subjects with depressive symptoms had an increased risk of liver-related mortality, and this association was stronger in HBsAg-positive participants than HBsAg-negative participants (P for interaction by HBsAg positivity = 0.036). Overall, after adjustment for potential confounders, HBsAg seropositivity, and family history of cancer, multivariable adjusted HR (95% CI) for liver-related mortality was 2.02 (1.12–3.66). Among HBsAg-positive subjects, multivariable-adjusted HR (95% CI) for liver-related mortality was 3.79 (1.66–8.67), whereas among HBsAg-negative subjects, corresponding HR (95% CI) was 1.23 (0.52–2.93) (Table 2). These patterns remained similar after further adjustment for FIB-4 and medication for liver disease, as well as for fatty liver and individual components of metabolic syndrome (blood pressure and cholesterol levels) (Supplementary Table 1), and also after further adjustment for antidepressant use (Supplementary Table 2). When changes in depressive symptoms and confounders during follow-up were treated as time-varying covariates, the results did not change qualitatively. Similarly, the association between depressive symptoms and liver cancer mortality tended to be stronger among HBsAg-positive participants even though the interaction according to HBsAg seropositivity was not statistically significant (P for interaction according to HBsAg positivity = 0.097) (Table 3).

**Table 2:** Hazard ratios (95% CIs) for liver-related mortality according to depressive symptoms by HBsAg positivity (*n* = 342,998).

CES-D score category	Person-years	Number of events	Mortality rate (per 10^5^ person-years)	Age-sex adjusted HR (95% CI)	Multivariable-adjusted HR^†^ (95% CI)		HR (95% CI) ^‡^ in model using time-dependent variables
					Model 1	Model 2	
**Total**							
< 16	1,613,583.7	60	3.7	1.00 (reference)	1.00 (reference)	1.00 (reference)	1.00 (reference)
≥ 16	222,924.8	14	6.3	2.24 (1.25–4.04)	2.02 (1.12–3.66)	2.00 (1.10–3.63)	2.46 (1.38–4.38)
**HBsAg (−)**							
< 16	1,559,740.4	39	2.5	1.00 (reference)	1.00 (reference)	1.00 (reference)	1.00 (reference)
≥ 16	216,366.8	6	2.8	1.45 (0.61–3.44)	1.23 (0.52–2.93)	1.16 (0.49–2.76)	1.78 (0.85–3.75)
**HBsAg (+)**							
< 16	53,843.2	21	39.0	1.00 (reference)	1.00 (reference)	1.00 (reference)	1.00 (reference)
≥ 16	6,558.0	8	122.0	3.75 (1.65–8.54)	3.79 (1.66–8.67)	4.22 (1.81–9.88)	4.50 (1.81–11.14)

*HBsAg* Hepatitis B surface antigen, *BMI* body mass index, *CI* confidence interval, *CVD* cardiovascular disease, *HR* hazard ratio, *FIB-4* Fibrosis-4 score, *CES-D* center for epidemiological studies-depression.

*P* = 0.036 for the overall interaction between HBsAg positivity and CES-D score category for liver-related mortality (multivariable model 2).

^†^Estimated from Cox proportional hazard models using age as a timescale to estimate hazard ratios (HRs) and 95 percent confidence intervals (95% CIs). Multivariable model 1 was adjusted for age (timescale), sex, center, year of screening exam, smoking status, alcohol consumption, total energy intake, physical activity, BMI, education level, history of diabetes, history of hypertension, history of CVD, HBsAg positivity (only for total subjects) and family history of cancer; model 2: model 1 plus adjustment for medication for liver disease and FIB-4.

^‡^Estimated from Cox proportional hazard models with CES-D score category, alcohol consumption, smoking status, physical activity, total energy intake, BMI, history of diabetes, history of hypertension, history of CVD, medication for liver disease and FIB-4 as time-dependent categorical variables and baseline age, sex, center, year of screening exam, education level, and family history of cancer as time-fixed variables.

**Table 3:** Hazard ratios (95% CI) for liver cancer mortality according to depressive symptoms by HBsAg positivity (*n* = 342,998).

CES-D score category	Person-years	Number of events	Mortality rate (per 10^5^ person-years)	Age-sex adjusted HR (95% CI)	Multivariable-adjusted HR^†^ (95% CI)		HR (95% CI) ^‡^ in model using time-dependent variables
					Model 1	Model 2	
**Total**							
< 16	1,613,583.7	44	2.7	1.00 (reference)	1.00 (reference)	1.00 (reference)	1.00 (reference)
≥ 16	222,924.8	10	4.5	2.18 (1.09–4.36)	2.07 (1.03–4.18)	2.19 (1.08–4.42)	1.88 (0.90–3.93)
**HBsAg (−)**							
< 16	1,559,740.4	23	1.5	1.00 (reference)	1.00 (reference)	1.00 (reference)	1.00 (reference)
≥ 16	216,366.8	3	1.4	1.22 (0.37–4.09)	1.11 (0.33–3.71)	1.08 (0.32–3.62)	0.91 (0.27–3.05)
**HBsAg (+)**							
< 16	53,843.2	21	39.0	1.00 (reference)	1.00 (reference)	1.00 (reference)	1.00 (reference)
≥ 16	6,558.0	7	106.7	3.22 (1.36–7.67)	3.31 (1.37–7.97)	3.82 (1.57–9.25)	3.83 (1.47–9.98)

*HBsAg* Hepatitis B surface antigen, *BMI* body mass index, *CI* confidence interval, *CVD* cardiovascular disease, *HR* hazard ratio, *FIB-4* Fibrosis-4 score, *CES-D* center for epidemiological studies-depression.

Note: *P* = *0.097* for the overall interaction between HBsAg positivity and CES-D score category for liver-related mortality (multivariable model).

^†^Estimated from Cox proportional hazard models using age as a timescale to estimate hazard ratios (HRs) and 95 percent confidence intervals (95% CIs). Multivariable model 1 was adjusted for age (timescale), sex, center, year of screening exam, smoking status, alcohol consumption, total energy intake, physical activity, BMI, education level, history of diabetes, history of hypertension, history of CVD, HBsAg positivity (only for total subjects) and family history of cancer; model 2: model 1 plus adjustment for medication for liver disease and FIB-4.

^‡^Estimated from Cox proportional hazard models with CES-D score category, alcohol consumption, smoking status, physical activity, total energy intake, BMI, history of diabetes, history of hypertension, history of CVD, medication for liver disease and FIB-4 as time-dependent categorical variables and baseline age, sex, center, year of screening exam, education level, and family history of cancer as time-fixed variables.

**Figure 1:**
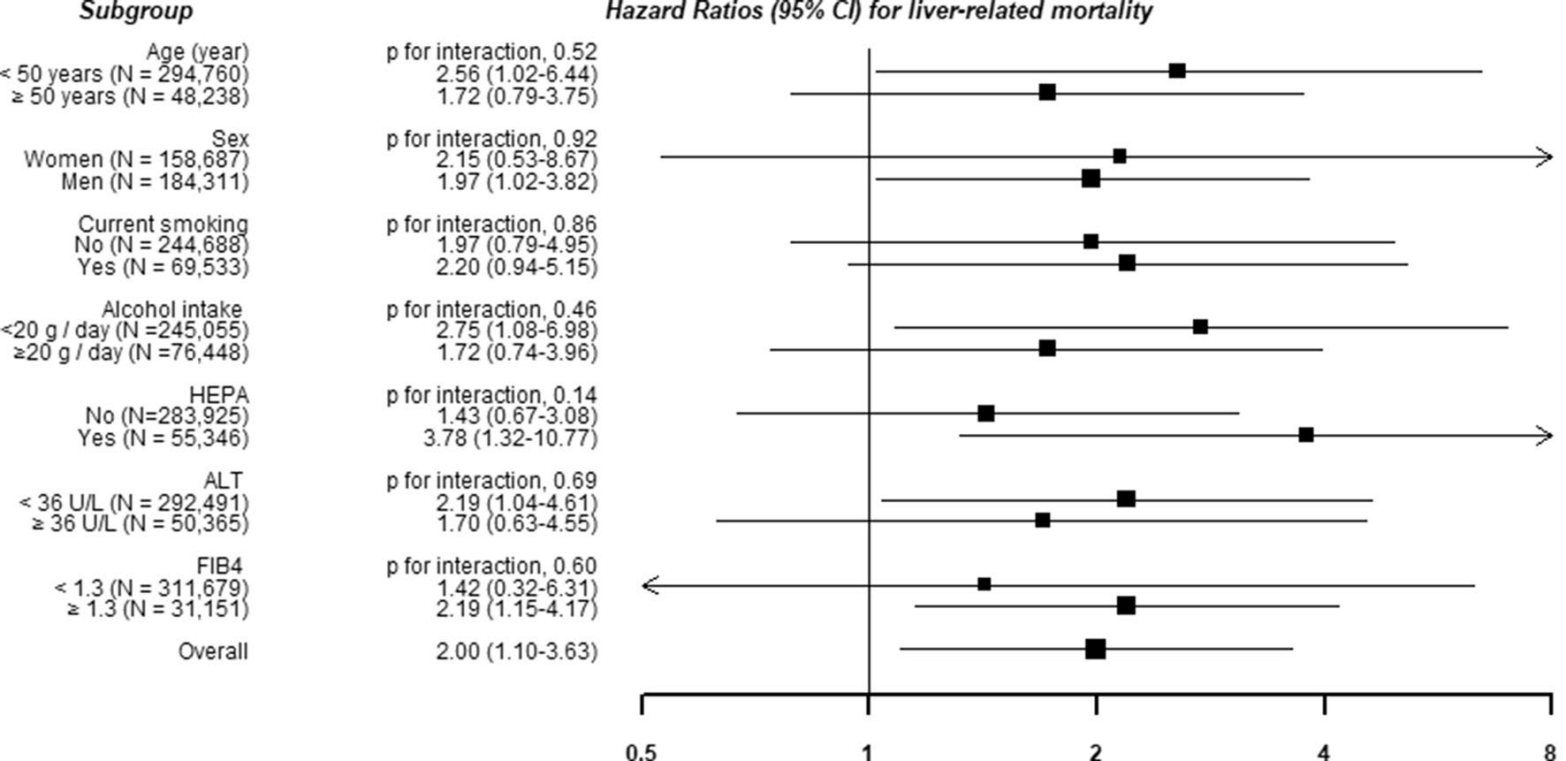
Hazard ratios (HRs) and 95% confidence intervals (CIs) for liver cancer mortality according to the presence of depressive symptoms in clinically relevant subgroups. Estimated from Cox proportional hazard models using age as a timescale to estimate HRs and 95% CIs. Multivariable model was adjusted for age (timescale), sex, center, year of screening exam, smoking status, alcohol consumption, total energy intake, physical activity, body mass index, education level, history of diabetes, history of hypertension, history of cardiovascular diseases, HBsAg positivity, family history of cancer, medication for liver disease, and Fibrosis-4 scores.

**Figure 2:**
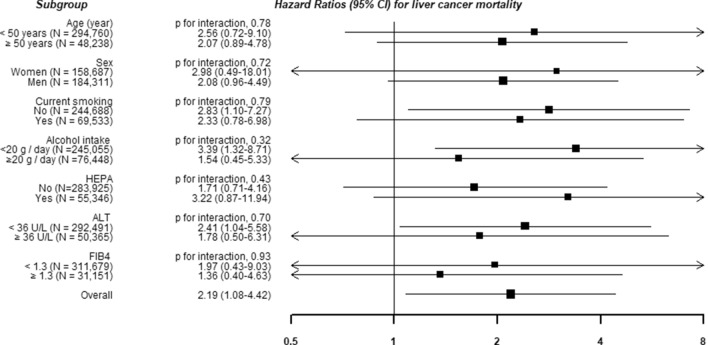
Hazard ratios (HRs) and 95% confidence intervals (CIs) for liver-related mortality according to the presence of depressive symptoms in clinically relevant subgroups. Estimated from Cox proportional hazard models using age as a timescale to estimate HRs and 95% CIs. Multivariable model was adjusted for age (timescale), sex, center, year of screening exam, smoking status, alcohol consumption, total energy intake, physical activity, body mass index, education level, history of diabetes, history of hypertension, history of cardiovascular diseases, HBsAg positivity, family history of cancer, medication for liver disease, and Fibrosis-4 scores.

Associations of depressive symptoms with liver-related mortality did not differ by clinically relevant subgroups (Figs. 1 and 2).

## Discussion

In a cohort of 342,998 young and middle-aged Korean individuals, depressive symptoms were positively and independently associated with liver-related mortality and liver cancer mortality. When performing analysis by HBsAg seropositivity, these associations were evident in only HBsAg-positive individuals. Furthermore, when changes in Center for Epidemiological Studies-Depression (CES-D) score category, medication for liver disease, fibrosis score, and other confounders during follow-up were updated as time-varying covariates, the association of depressive symptoms with liver cancer and liver-related mortality remained. Our findings indicate that depressive symptoms appear to be associated with increased risk of liver cancer and liver-related mortality, especially in HBsAg-positive individuals.

Several studies have addressed the impact of depression on mortality risk in patients with liver cirrhosis or cancer and liver transplant recipients^11,12^, and reported the negative effect of depressive symptoms on survival. However, few studies have investigated the relationship between depression and liver-related mortality in non-patient populations, with conflicting results. A meta-analysis using general population-based studies showed that psychological distress as assessed by the 12-item version of the General Health Questionnaire (GHQ-12) was associated with liver disease mortality^13^. On the contrary, other studies using administrative claims databases have reported no association between depression and liver cancer^15–17^ and even an inverse association between depression and liver disease mortality^14^. However, those studies were limited by inclusion of only severely depressive patients requiring admission^15,16^, inclusion of only elderly patients over 65 years old^17^, and lack of consideration of important covariates such as smoking, alcohol intake, physical activity, and medication for liver disease^15–17^.

Our study findings are in line with a recent meta-analysis that found a small and positive association between depression and liver cancer risk with a corresponding relative risk (95% CI) of 1.20 (1.01–1.43)^18^. Our study involving a young and middle-aged and relatively healthy population for whom a range of covariates were available, enabled us to examine the independent relationships of depressive symptoms with liver cancer and liver-related mortality in HBsAg-negative and HBsAg-positive individuals while accounting for depressive symptoms and confounding factors at both baseline and follow-up. Our study results are less likely to be affected by patient selection bias or by biases related to comorbidities than findings from previous studies of older individuals or specific patient samples; thus, our findings may be more generalizable for low-risk populations. Furthermore, our assessment of depressive symptoms was more inclusive because we used a standardized instrument to define depressive symptoms compared to analysis of diagnosis codes in claims databases or admission for depression.

Besides depression, other factors may also affect liver-related mortality and liver cancer mortality. HBV^19^, nonalcoholic fatty liver disease^19^, metabolic syndrome and its components^20,21^ have also been associated with increased risk for liver cancer. In addition, previous studies have reported a higher risk of HCC in chronic hepatitis B patients with metabolic syndrome, or metabolic risk factors^22^. Meanwhile, psychotropic medication such as antidepressants may impact metabolic risk factors such as obesity, dyslipidemia, insulin resistance, and blood pressure^23^. The relationship between depressive symptoms and metabolic health may also be bidirectional^24,25^, but a recent study only found evidence for a relationship between depressive symptoms and subsequent metabolic health^26^. Patients with liver disease may also be more susceptible to depression^27^, but the opposite relationship has also been reported^28^. However, the association of depressive symptoms with liver-related mortality and liver cancer mortality observed in our study remained significant even after adjustment for several covariates, including metabolic components, antidepressant use, medication for liver disease, and fibrosis scores.

The exact mechanisms underlying the association between depression and liver cancer and liver-related mortality are not fully understood. Depression might increase mortality through alteration of the immune system. Evidence suggests that depression can cause impairment of the immune system and initiation or progression of cancer in association with a deoxyribonucleic acid (DNA) tumor virus^29^. HBV is a DNA virus, which might explain why HBsAg-positive but not HBsAg-negative individuals who were depressed were at higher risk of liver cancer mortality. Additionally, both HBV infection and depression can be associated with chronic inflammation^30–32^ and coexistence of both conditions might aggravate such inflammation, causing subsequent liver damage. Activation of the hypothalamus–pituitary–adrenal axis in depression may also result in production of mediators that suppress stimulation of natural killer (NK)-cell activity, phagocytosis, cytotoxic T-cell activity, and the production of inflammatory cytokines^29^. A study on hepatitis-B and C-related HCC patients also demonstrated that patients with depressive symptoms had lower NK cell numbers than those without depressive symptoms, and because a decline in NK cell number was correlated with survival time, NK cell number was hypothesized to be an intermediary step for mortality risk^12,33^.

Meanwhile, other researchers have recommended caution when interpreting results from studies on depression and survival, suggesting that depression may be a risk marker, but not a causal risk factor^34^. Previous studies have reported associations between self-reported depression and mortality for a wide range of health problems, but some studies have shown that these associations disappeared after considering appropriate control variables^35,36^. People with depressive symptoms can be at increased risk of unhealthy lifestyle behaviors including excessive alcohol drinking, smoking, substance abuse, inadequate physical activity and obesity; thus, depression may affect mortality through its negative effects on health behaviors^36,37^. Exercise has also been suggested to improve depressive symptoms^38^, while smoking^39^ and alcohol consumption^40^ have shown a potential association with subsequent depression. Depressive symptoms might also negatively affect use of healthcare services and compliance with treatment, and therefore adversely affect health outcomes. However, in our study, even after adjustment for smoking, alcohol intake, physical activity, medication for liver disease, and fibrosis score at both baseline and follow-up visits, the relationship of depressive symptoms with liver-related mortality and liver cancer mortality was consistent, and the associations did not differ in subgroup analysis; thus, behavioral factors could not fully explain the observed associations between depressive symptoms and liver cancer mortality or liver-related mortality.

Currently, multiple organizations recommend routine screening for depression in patients at high risk, including individuals with chronic medical conditions^41^. Previous research suggests that psychosocial interventions may improve immune system functioning and survival^42^, and a recent study also revealed that use of selective serotonin reuptake inhibitors, which are antidepressants, may reduce HCC risk in patients with HBV infection^43^. However, further research is required to confirm these results and to determine whether screening and treatment for depression may improve liver-related outcomes in HBsAg-positive patients.

Our study had several limitations. First, depressive symptoms were assessed through CES-D, a validated assessment tool, but not a clinical diagnosis of depression. However, the CES-D is recommended when depression is the sole focus of the study and is widely used in population-based research^44^. Second, information on liver cancer incidence was not available. Given that the 5-year relative survival of liver cancer is among the lowest of various cancer types directly after pancreatic cancer (18%), a similar association between depression and liver cancer incidence might be expected^45^. However, further studies are required to investigate the relationship between depressive symptoms and liver cancer incidence. Third, chronic hepatitis B is characterized by several phases: an immune tolerant phase, an immune active phase, and an inactive phase, and these phases can have different effects on liver-related outcomes^46^. Unfortunately, information on HBV DNA, hepatitis B e-antigen (HBeAg), and antibodies to HBeAg were not available in this study. Fourth, information on past medical history and health behaviors were also based on self-administered questionnaires, and some degree of measurement error is therefore inevitable. Additionally, we cannot exclude the possibility that we did not consider some unmeasured or residual confounding factors associated with depressive symptoms and liver-related mortality. Finally, our study included mostly middle-aged, relatively healthy Koreans undergoing routine health examinations, and therefore our findings may not be generalizable to older groups or other ethnicities.

In this large cohort study, depressive symptoms were independently associated with an increased risk of liver-related mortality and liver cancer mortality, especially in HBsAg-positive individuals. Further research is required to determine whether screening and treatment for depression may benefit liver-related outcomes, including the incidence of liver cancer, in HBsAg-positive patients.

## Methods

### Study population

The Kangbuk Samsung Health Study is a cohort including men and women who received a comprehensive health examination every one or two years at one of the Kangbuk Samsung Hospital Total Healthcare Centers in Seoul or Suwon, South Korea^47,48^. In Korea, the Industrial Safety and Health Law mandates health screening exams for employees every one to two years. Over 80% of the participants were employees or their spouses. The remaining voluntarily purchased and participated in the health checkup program. Our study population comprised examinees who participated in comprehensive health screening exams between 2011 and 2016 (n = 418,214). This study used data routinely collected during the health screening assessments, which include questionnaires, blood tests, imaging exams, and procedures (e.g., ultrasound)^47^.

**Figure 3:**
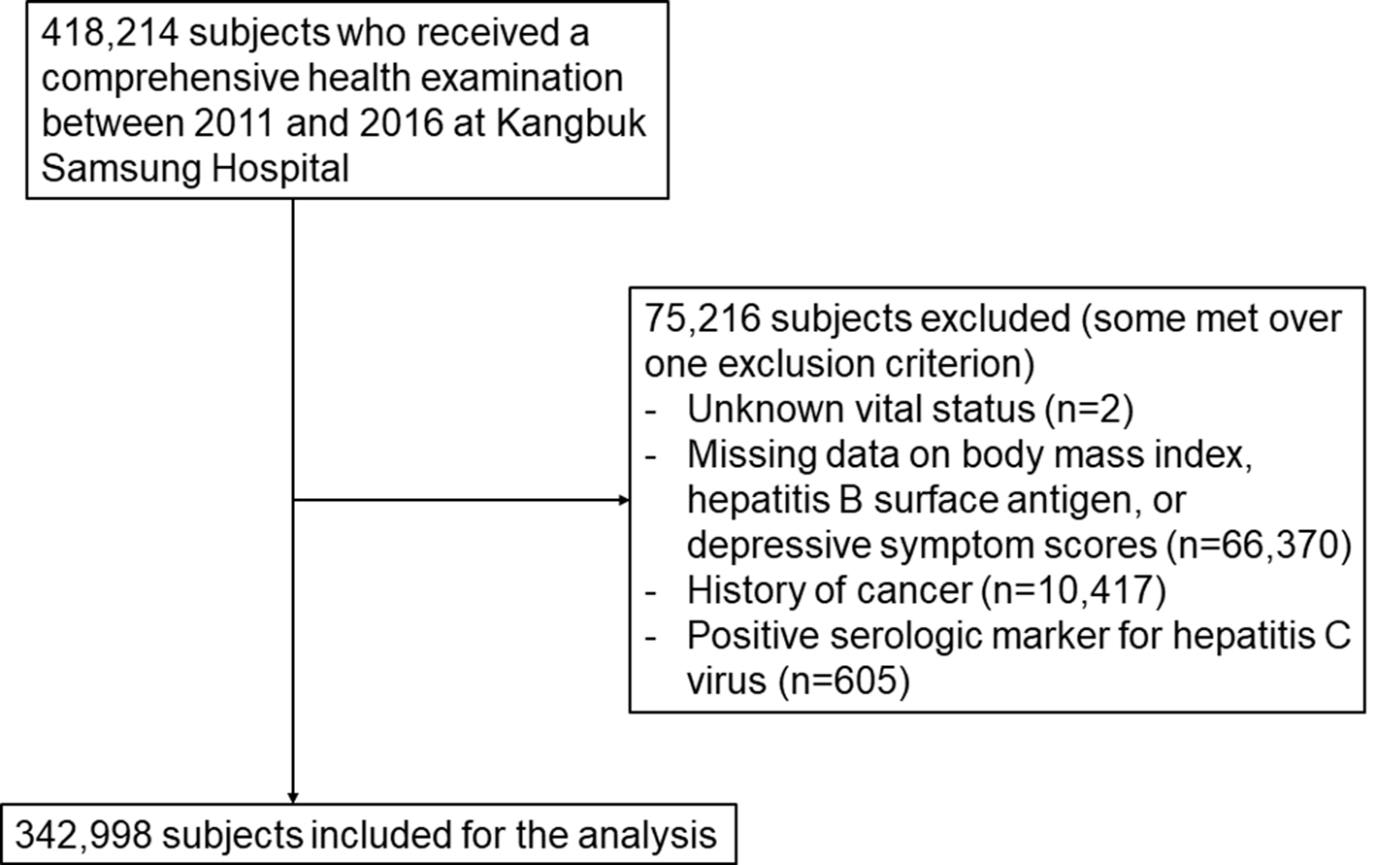
Flowchart of study participants.

We excluded 75,216 subjects who met one or more of the following exclusion criteria at baseline (Fig. 3): unknown vital status (n = 2); missing data on body mass index (BMI), HBsAg, or depressive symptoms (see below for further details) (n = 66,370); a history of cancer (n = 10,417); and/or positive for serologic markers for hepatitis C virus (n = 605)^49^. Some participants met more than one exclusion criterion, thus a total of 342,998 participants were included in the analysis. This study was approved by the Institutional Review Board of Kangbuk Samsung Hospital (IRB 2020-03-011), and was performed in accordance with the 1964 Declaration of Helsinki and its later amendments. The requirement for informed consent was waived due to our use of a preexisting de-identified dataset that combined data routinely collected during the health screening process and mortality data (see below for further details).

### Data collection

All the tests were performed at the Kangbuk Samsung Hospital Health Screening Center clinics in Seoul or Suwon. A standardized, self-administered questionnaire was used to collect data on demographic characteristics, health behaviors, medical history, and medications. Current smoking status was classified as never, former, or current smokers. Current alcohol consumption was assessed by the frequency of alcohol consumption per week and the amount consumed per drinking day. Average alcohol consumption per day was based on the frequency and amount of alcohol consumed per drinking day. Physical activity levels were determined using the Korean validated version of the International Physical Activity Questionnaire (IPAQ) Short Form, and were categorized into inactive, minimally active, or health-enhancing physical activity (HEPA)^50,51^. HEPA was defined by physical activity that met either of the following criteria: (1) activity of vigorous intensity performed three or more days per week to accumulate ≥ 1500 metabolic equivalent (MET)-minutes/week (1 MET is energy expenditure at rest); or (2) seven days of walking, moderate or vigorous intensity activities that amount to at least 3000 MET min/week^51^. Usual dietary consumption over the past year was assessed using a food frequency questionnaire validated for use in South Korea^52^. A family history of cancer was defined as having one or more first-degree relatives with any type of cancer disclosed in a self-reported questionnaire.

The Korean version of the 20-item CES-D scale was used to evaluate depressive symptoms: the internal consistency of the Korean version has been reported to range from 0.84 to 0.91^53^. The presence of clinically significant depressive symptoms was determined by a CES-D score ≥ 16. This cut-off was established and validated through previous studies^54,55^. A history of depression was defined as physician-diagnosed depression and antidepressant use in the last month as assessed via self-administered questionnaire.

Trained nurses measured sitting blood pressure (BP), height, and weight. Obesity was defined as a body mass index (BMI) ≥ 25 kg/m^2^, which is the proposed cut-off for diagnosing obesity in Asians^56^. Hypertension was defined by systolic BP ≥ 140 mmHg, diastolic BP ≥ 90 mmHg, or the use of antihypertensive medications.

### Laboratory analyses

Fasting blood tests included aspartate aminotransferase (AST), alanine aminotransferase (ALT), gamma-glutamyltransferase (GGT), total cholesterol, low-density lipoprotein cholesterol (LDL-C), high-density lipoprotein cholesterol (HDL-C), triglycerides, glucose, insulin, albumin, platelets, hepatitis B surface antigen (HBsAg), and HCV antibodies (HCV Ab) as previously described^47^. Hepatitis B serologic testing was performed by electrochemiluminescent immunoassay (Modular E170; Roche Diagnostics, Tokyo, Japan). The identification of HBsAg at the first testing was interpreted to indicate chronic HBV infection. Insulin resistance was assessed using the HOMA-IR equation as follows: fasting blood insulin (μU/mL) × fasting blood glucose (mmol/l)/22.5. Diabetes mellitus (DM) was defined as a fasting serum glucose level ≥ 126 mg/dL, hemoglobin A1c (HbA1c) ≥ 6.5%, or current use of anti-diabetic medications.

Fatty liver was diagnosed based on abdominal ultrasounds performed by experienced radiologists who were blind to the aims of our study, and was based on standard criteria, including a diffuse increase of fine echoes in the liver parenchyma in comparison to the kidney or spleen, deep beam attenuation, and also bright vessel walls^57^. Liver cirrhosis was defined by changes in liver volume distribution, surface nodularity, accentuation of the fissure, heterogeneity, cirrhotic nodules, bright and coarsened hepatic architecture, and signs of portal hypertension^58^.

FIB-4 score, which is a non-invasive fibrosis index, was used to categorize the probability of liver fibrosis. FIB-4 score was calculated using the following formula: FIB-4 = (age (years) × AST (U/L))/(platelet count (× 10^9^/L) × ALT (U/L)^1/2^). Cut-off values were used to define low (FIB-4 < 1.30), intermediate (FIB-4, 1.30− < 2.67), and high (FIB-4 ≥ 2.67) probability of advanced fibrosis^59^.

### Mortality follow-up

Mortality follow-up was based on nationwide death certificate data provided by the Korean National Statistical Office, until the end of 2018. Death certificate data for Korean adults are practically complete, because the Korean National Statistical Office receives notice of all deaths that occur. We determined the cause of death by the underlying cause recorded on each death certificate, following the International Classification of Diseases and Related Health Problems 10th Revision (ICD-10). Concordance between the death certificates’ cause of death and diagnosis in medical utilization data was 91.9% for all-cause deaths, and 94.9% for deaths due to cancer^60,61^. Liver cancer mortality included death due to malignant neoplasm of the liver and the intrahepatic bile ducts (ICD-10 C22)^62^. Liver-related mortality encompassed deaths due to chronic liver disease and cirrhosis (ICD-10 K70, K73-74), and also malignant neoplasm of the liver and the intrahepatic bile ducts (ICD-10 C22)^62^.

### Statistical analysis

Because there was a difference in age and sex between those with depressive symptoms and those without, all baseline characteristics are presented as age- and sex-adjusted means or proportions with 95% confidence intervals (CIs).

The primary endpoint was the development of liver-related mortality or the development of liver cancer-related mortality. Participants were followed from their baseline exam until either the development of the endpoint or the end of 2018, whichever came first. Those who died due to other causes were censored at their date of death. Hazard ratios (HRs) and 95% CIs for liver-related or liver cancer mortality were calculated using Cox proportional hazards regression analyses. Age was used as the timescale, and was documented as the age at which subjects received their first health checkup exam (left truncation) and the age at which they exited the analysis on their date of death, or on December 31, 2018. This approach controls for age in our analysis. The proportional hazards assumption was evaluated by inspecting graphs of the estimated log(− log (*SURVIVAL*)). We found no major violations of the proportional hazards assumption.

The risks of liver-related mortality and liver cancer mortality were evaluated separately according to the presence of depressive symptoms. Models were initially adjusted for age (timescale) and sex and then further adjusted for additional potential confounders including study center (Seoul or Suwon), year of screening exam, smoking (never, past, current, or unknown), alcohol intake (none, < 20 g/day, or ≥ 20 g/day, unknown), physical activity, BMI, education level (< community college graduate, ≥ community college graduate, or unknown), family history of cancer, total energy intake, history of diabetes, history of hypertension, history of CVD, and HBsAg (Model 1). Model 2 was further adjusted for FIB-4 and medication for liver disease. Additionally, we conducted time-dependent analyses where changes in depressive symptoms and other covariates during follow-up were updated as time-varying covariates in the models.

Subgroup analyses were performed by age (< 50 vs. ≥ 50 years), sex (men vs. women), smoking status (never smokers vs. ever smokers), alcohol intake (< 20 vs. ≥ 20 g/day), HEPA (no vs. yes), BMI (< 25 vs. ≥ 25 kg/m^2^), DM (no vs. yes), ALT (< 36 vs. ≥ 36 U/l), and noninvasive fibrosis score based on FIB-4 (low vs. intermediate or high probability). Interactions between depressive symptoms and subgroup characteristics were tested using likelihood ratio tests that compared models with or without multiplicative interaction terms. Statistical analyses were performed using STATA version 16.0 (StataCorp LP, College Station, TX, USA). All reported P values are two-tailed. Differences with a *P* value < 0.05 were considered statistically significant.

## Supplementary information

Supplementary Information.

## Data Availability

The data are not available to be shared publicly because we do not have a permission from the IRB to distribute the data.
