# Supplementary material for: Depressive symptoms and risk of liver-related mortality in individuals with hepatitis B virus infection: a cohort study
Source: Sci Rep. 2020 Nov 30;10:20812. doi: 10.1038/s41598-020-77886-2 (PMC7705706; doi:10.1038/s41598-020-77886-2)
Supplement: Supplementary file 1 — Supplementary Information. [file 41598_2020_77886_MOESM1_ESM.docx]

**Depressive symptoms and risk of liver-related mortality in individuals with hepatitis B virus infection: A cohort study**

In Young Cho^1,†^, Yoosoo Chang^2,3,4,†^, Eunju Sung^1,2^; Won Sohn^5^, Jae-Heon Kang^1^; Hocheol Shin^1,2,*^, Seungho Ryu^2,3,4,*^

^1^Department of Family Medicine, Kangbuk Samsung Hospital, Sungkyunkwan University School of Medicine, Seoul, South Korea

^2^Center for Cohort Studies, Total Healthcare Center, Kangbuk Samsung Hospital, Sungkyunkwan University School of Medicine, Seoul, South Korea

^3^Department of Occupational and Environmental Medicine, Kangbuk Samsung Hospital, Sungkyunkwan University School of Medicine, Seoul, South Korea

^4^Department of Clinical Research Design & Evaluation, SAIHST, Sungkyunkwan University, Seoul, South Korea

^5^Division of Gastroenterology, Department of Internal Medicine, Kangbuk Samsung Hospital, Sungkyunkwan University School of Medicine, Seoul, South Korea

**^†^Drs Cho and Chang contributed equally as co-first authors**

**^*^Drs Shin and Ryu are corresponding authors.**

**^*^Correspondence:**

Hocheol Shin, Department of Family Medicine, Kangbuk Samsung Hospital, Sungkyunkwan University School of Medicine, 29 Saemunan-ro, Jongno-gu, Seoul 03181, Korea.

Tel: +82-2-2001-2001; Fax: +82-2-2001-2016; E-mail: [hcfm.shin@samsung.com](mailto:hcfm.shin@samsung.com).

**and**

Seungho Ryu, Department of Occupational and Environmental Medicine, Kangbuk Samsung Hospital, Sungkyunkwan University School of Medicine, Samsung Main Building B2, 250 Taepyung-ro 2ga, Jung-gu, Seoul 04514, Korea.

Tel: +82-2-2001-5137; Fax: +82-2-757-0436; E-mail: [sh703.yoo@gmail.com](mailto:sh703.yoo@gmail.com).

**Supplementary Table 1.** Hazard ratios (95% CIs) for liver-related or liver cancer mortality according to depressive symptoms by HBsAg positivity (*n* = 342,998) with further adjustment for fatty liver and components of metabolic syndrome (cholesterol levels and blood pressure).

| CES-D score category | Multivariable-adjusted HR^†^ (95% CI) | |
| --- | --- | --- |
|  | For liver-related mortality | For liver cancer mortality |
| Total |  |  |
| <16 | 1.00 (reference) | 1.00 (reference) |
| ≥16 | 1.91 (1.03-3.56) | 2.32 (1.14-4.72) |
| HBsAg (-) |  |  |
| <16 | 1.00 (reference) | 1.00 (reference) |
| ≥16 | 1.21 (0.51-2.88) | 1.15 (0.34-3.88) |
| HBsAg (+) |  |  |
| <16 | 1.00 (reference) | 1.00 (reference) |
| ≥16 | 3.81 (1.55-9.34) | 4.05 (1.66-9.89) |

Note: *P =* 0.07 for the overall interaction between HBsAg positivity and CES-D score category for liver-related mortality; *P =* 0.101 for the overall interaction between HBsAg positivity and CES-D score category for liver-cancer mortality.

^†^Estimated from Cox proportional hazard models using age as a timescale to estimate hazard ratios (HRs) and 95% confidence intervals (95% CIs). Multivariable model 1 was adjusted for age (timescale), sex, center, year of screening exam, smoking status, alcohol consumption, total energy intake, physical activity, BMI, education level, history of diabetes, history of hypertension, history of CVD, HBsAg positivity (only for total subjects) and family history of cancer, medication for liver disease, FIB-4 and fatty liver, total cholesterol, HDL-cholesterol, triglyceride, glucose and systolic blood pressure.

**Supplementary Table 2** Hazard ratios (95% CIs) for liver-related or liver cancer mortality according to depressive symptoms by HBsAg positivity after further adjustment for anti-depressants.

| CES-D score category | Multivariable-adjusted HR^†^ (95% CI) | |
| --- | --- | --- |
|  | For liver-related mortality | For liver cancer mortality |
| Total |  |  |
| <16 | 1.00 (reference) | 1.00 (reference) |
| ≥16 | 1.91 (1.05-3.49) | 2.11 (1.04-4.29) |
| HBsAg (-) |  |  |
| <16 | 1.00 (reference) | 1.00 (reference) |
| ≥16 | 1.06 (0.44-2.56) | 1.01 (0.30-3.42) |
| HBsAg (+) |  |  |
| <16 | 1.00 (reference) | 1.00 (reference) |
| ≥16 | 4.28 (1.83-10.00) | 3.78 (1.56-9.16) |

Note: *P =* 0.07 for the overall interaction between HBsAg positivity and CES-D score category for liver-related mortality; *P =* 0.084 for the overall interaction between HBsAg positivity and CES-D score category for liver-cancer mortality.

^†^Estimated from Cox proportional hazard models using age as a timescale to estimate hazard ratios (HRs) and 95 percent confidence intervals (95% CIs). Multivariable model 1 was adjusted for age (timescale), sex, center, year of screening exam, smoking status, alcohol consumption, total energy intake, physical activity, BMI, education level, history of diabetes, history of hypertension, history of CVD, HBsAg positivity (only for total subjects) and family history of cancer, medication for liver disease, FIB-4 and anti-depressants.

**Supplementary Table 3** Hazard ratios (95% CIs) for liver-related or liver cancer mortality according to CESD domain by HBsAg positivity.

| CESD domain | Multivariable-adjusted HR^†^ (95% CI) | |
| --- | --- | --- |
|  | For liver-related mortality | For liver cancer mortality |
| Somatic domain |  |  |
| Total |  |  |
| <75th percentile | 1.00 (reference) | 1.00 (reference) |
| ≥75th percentile | 1.26 (0.69-2.30) | 1.48 (0.73-3.01) |
| HBsAg (-) |  |  |
| <75th percentile | 1.00 (reference) | 1.00 (reference) |
| ≥75th percentile | 0.92 (0.41-2.09) | 1.02 (0.35-3.01) |
| HBsAg (+) |  |  |
| <75th percentile | 1.00 (reference) | 1.00 (reference) |
| ≥75th percentile | 1.98 (0.82-4.79) | 3.78 (0.82-5.30) |
| P for interaction | 0.207 | 0.321 |
| Negative domain |  |  |
| Total |  |  |
| <75th percentile | 1.00 (reference) | 1.00 (reference) |
| ≥75th percentile | 0.99 (0.56-1.79) | 1.21 (0.63-2.35) |
| HBsAg (-) |  |  |
| <75th percentile | 1.00 (reference) | 1.00 (reference) |
| ≥75th percentile | 0.72 (0.32-1.63) | 1.00 (0.37-2.67) |
| HBsAg (+) |  |  |
| <75th percentile | 1.00 (reference) | 1.00 (reference) |
| ≥75th percentile | 1.53 (0.66-3.58) | 1.45 (0.59-3.54) |
| P for interaction | 0.208 | 0.578 |
| Anhedonia domain |  |  |
| Total |  |  |
| <75th percentile | 1.00 (reference) | 1.00 (reference) |
| ≥75th percentile | 1.39 (0.86-2.29) | 1.79 (1.02-3.16) |
| HBsAg (-) |  |  |
| <75th percentile | 1.00 (reference) | 1.00 (reference) |
| ≥75th percentile | 1.18 (0.63-2.21) | 1.59 (0.72-3.54) |
| HBsAg (+) |  |  |
| <75th percentile | 1.00 (reference) | 1.00 (reference) |
| ≥75th percentile | 1.83 (0.85-3.92) | 2.01 (0.92-4.39) |
| P for interaction | 0.377 | 0.679 |

^†^Estimated from Cox proportional hazard models using age as a timescale to estimate hazard ratios (HRs) and 95% confidence intervals (95% CIs). Multivariable model 1 was adjusted for age (timescale), sex, center, year of screening exam, smoking status, alcohol consumption, total energy intake, physical activity, BMI, education level, history of diabetes, history of hypertension, history of CVD, HBsAg positivity (only for total subjects) and family history of cancer, medication for liver disease, FIB-4 and anti-depressants.

**Supplementary Table 4** Hazard ratios (95% CIs) for liver-related or liver cancer mortality according to depressive symptoms by HBsAg positivity.

| CES-D score category | Multivariable-adjusted HR^†^ (95% CI) for liver-related mortality | | Multivariable-adjusted HR^†^ (95% CI) for liver cancer mortality | |
| --- | --- | --- | --- | --- |
|  | <40 years | $\geq$40 years | <40 years | $\geq$40 years |
| Number | 203,371 | 139,627 | 203,371 | 139,627 |
| Total |  |  |  |  |
| <16 | 1.00 (reference) | 1.00 (reference) | 1.00 (reference) | 1.00 (reference) |
| ≥16 | 1.66 (0.19-14.28) | 2.03 (1.09-3.77) | - | 2.29 (1.13-4.64) |
| HBsAg (-) |  |  |  |  |
| <16 | 1.00 (reference) | 1.00 (reference) | 1.00 (reference) | 1.00 (reference) |
| ≥16 | - | 1.26 (0.52-3.04) | - | 1.17 (0.35-3.97) |
| HBsAg (+) |  |  |  |  |
| <16 | 1.00 (reference) | 1.00 (reference) | 1.00 (reference) | 1.00 (reference) |
| ≥16 | 10.91 (0.66-179.43) | 3.70 (1.48-9.27) | - | 3.73 (1.49-9.34) |

^†^Estimated from Cox proportional hazard models using age as a timescale to estimate hazard ratios (HRs) and 95 percent confidence intervals (95% CIs). Multivariable model 1 was adjusted for age (timescale), sex, center, year of screening exam, smoking status, alcohol consumption, total energy intake, physical activity, BMI, education level, history of diabetes, history of hypertension, history of CVD, HBsAg positivity (only for total subjects) and family history of cancer, medication for liver disease, and FIB-4.
